# Dissecting endothelial cell heterogeneity with new tools

**DOI:** 10.1186/s13619-025-00223-3

**Published:** 2025-03-23

**Authors:** Jing Zhong, Rong-rong Gao, Xin Zhang, Jia-xin Yang, Yang Liu, Jinjin Ma, Qi Chen

**Affiliations:** 1 Center for Cell Lineage Atlas, CAS Key Laboratory of Regenerative Biology, Joint School of Life Sciences, Guangzhou Institutes of Biomedicine and Health, Chinese Academy of Sciences, Guangzhou Medical University, Guangzhou, 510530 China; 2 University of Chinese Academy of Sciences, Beijing, China; 3 China-New Zealand Belt and Road Joint Laboratory on Biomedicine and Health, Guangdong Provincial Key Laboratory of Stem Cell and Regenerative Medicine, Guangdong-Hong Kong Joint Laboratory for Stem Cell and Regenerative Medicine, Guangzhou Institutes of Biomedicine and Health, Chinese Academy of Sciences, Guangzhou, 510530 China; 4 Center for Cell Lineage Atlas, Guangzhou Institutes of Biomedicine and Health, Chinese Academy of Sciences, Guangzhou, 510530 China; 5 Biomedical Sciences College & Shandong Medicinal Biotechnology Centre, Shandong First Medical University & Shandong Academy of Medical Sciences, NHC Key Laboratory of Biotechnology Drugs (Shandong Academy of Medical Sciences); Key Lab for Rare & Uncommon Diseases of Shandong Province, Ji’nan 250117, Shandong, China; 6 The Innovation Centre of Ministry of Education for Development and Diseases, School of Medicine, South China University of Technology, Guangzhou, 510006 China; 7 The Institute of Future Health, South China of Technology, Guangzhou International Campus, Guangzhou, 511442 China

**Keywords:** Angiogenesis, Endothelial cell heterogeneity, Organotypic blood vessel, ScRNA-seq, Organoid

## Abstract

The formation of a blood vessel network is crucial for organ development and regeneration. Over the past three decades, the central molecular mechanisms governing blood vessel growth have been extensively studied. Recent evidence indicates that vascular endothelial cells—the specialized cells lining the inner surface of blood vessels—exhibit significant heterogeneity to meet the specific needs of different organs. This review focuses on the current understanding of endothelial cell heterogeneity, which includes both intra-organ and inter-organ heterogeneity. Intra-organ heterogeneity encompasses arterio-venous and tip-stalk endothelial cell specialization, while inter-organ heterogeneity refers to organ-specific transcriptomic profiles and functions. Advances in single-cell RNA sequencing (scRNA-seq) have enabled the identification of new endothelial subpopulations and the comparison of gene expression patterns across different subsets of endothelial cells. Integrating scRNA-seq with other high-throughput sequencing technologies promises to deepen our understanding of endothelial cell heterogeneity at the epigenetic level and in a spatially resolved context. To further explore human endothelial cell heterogeneity, vascular organoids offer powerful tools for studying gene function in three-dimensional culture systems and for investigating endothelial-tissue interactions using human cells. Developing organ-specific vascular organoids presents unique opportunities to unravel inter-organ endothelial cell heterogeneity and its implications for human disease. Emerging technologies, such as scRNA-seq and vascular organoids, are poised to transform our understanding of endothelial cell heterogeneity and pave the way for innovative therapeutic strategies to address human vascular diseases.

**Graphical Abstract:**

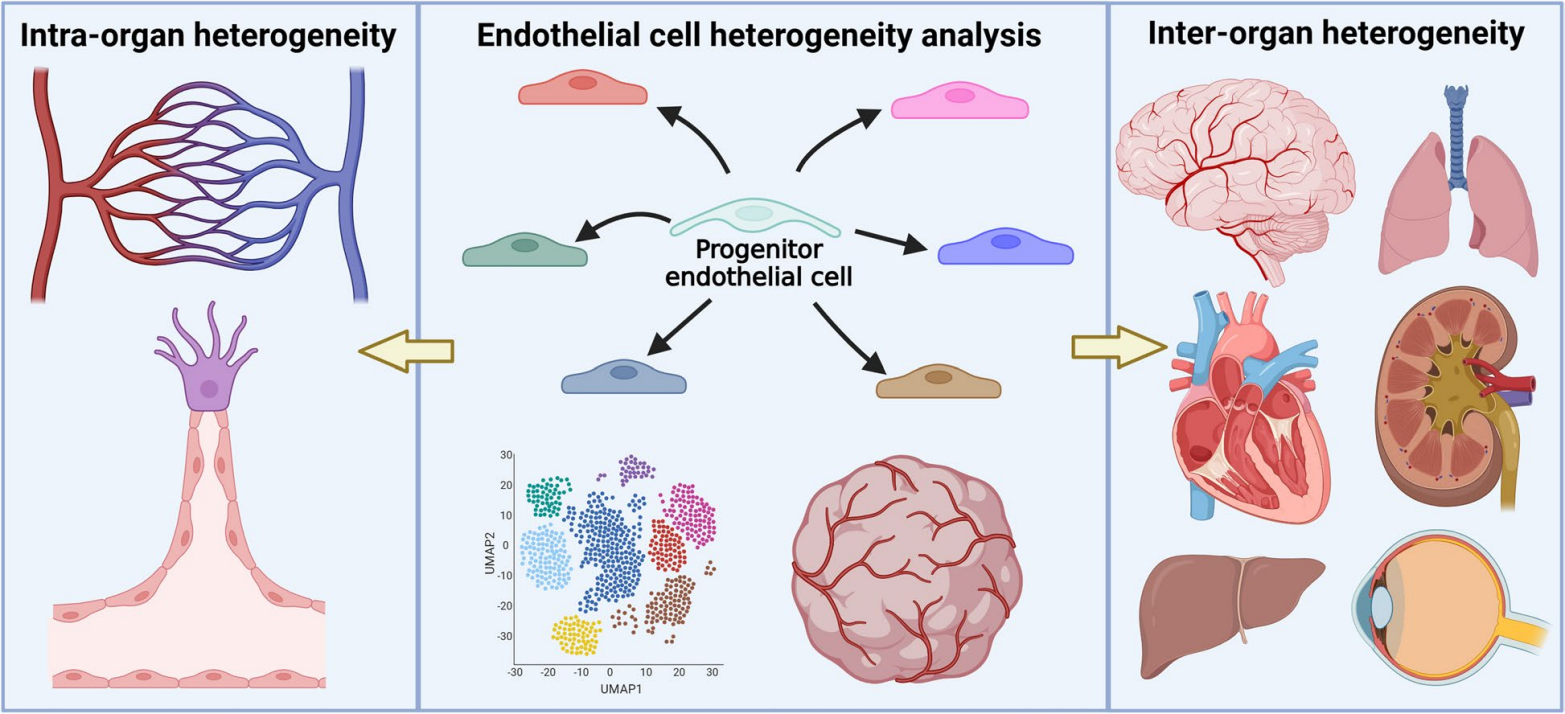

## Background

**Fig. 1:**
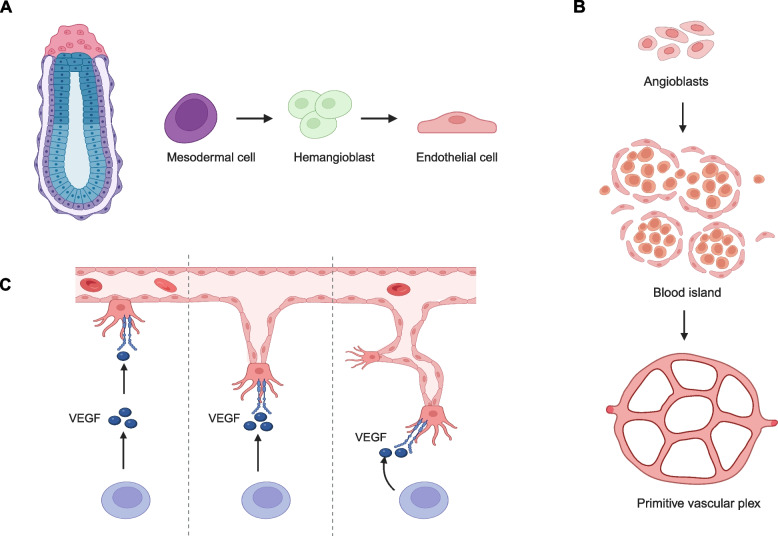
Developmental procedure for vascular development. **A** Endothelial cells are differentiated from mesodermal cell through hemangioblast. **B** The cellular process of vasculogenesis. Angioblast cells fuse into blood islands, which connect to form the primitive vascular plexus. **C** The cellular process of angiogenesis, in which hypoxia induce tissue resident cells release vascular endothelial cell growth factor (VEGF) to induce growth of blood vessel toward hypoxia region

Blood vessels constitute the foundation of our circulatory system, which is vital for nutrient transport, immune surveillance, and organ development. The vascular development can be categorized into vasculogenesis and angiogenesis, whose molecular mechanisms have been extensively investigated in the last three decades. Vasculogenesis refers to the de novo formation of primitive vascular plexus. The mesodermal progenitors differentiate into vascular endothelial cells (VEC) (Fig. 1A), which assemble into the primitive blood islands that expand and gradually polarize to form the vascular tube (Fig. 1B) (Krüger-Genge et al. 2019). From this primitive vascular plexus, VEC undergo a complex cellular process termed angiogenesis to form new blood vessels from pre-existing ones (Makanya et al. 2009). In brief, tissues lacking oxygen result in up-regulation of hypoxia-inducible factor signaling, which release vascular endothelial cell growth factor A (VEGF-A) to attract growth of blood vessels toward hypoxia region (Fig. 1C). The process of sprouting angiogenesis requires disruption of extracellular matrix, selection of angiogenic (tip) endothelial cells, formation of vascular tube and anastomosis, which are summarized in other outstanding reviews (Apte et al. 2019; Blanco and Gerhardt 2013; Chen et al. 2019b; Marziano et al. 2021). As highly branched and functional system, the body’s vascular network is connected and remodeled into morphologically, genetically, and functionally distinct networks of arteries, veins, and capillaries. The differentiation of VEC occurs at virtually all stages of vascular development in an organism, from the emergence of the primitive vascular plexus to the establishment of a functional circulatory system in response to physiological and pathological changes in the organism.

During development and differentiation of the organism, endothelial cells deviate from the original homogeneous population in order to transform into a heterogeneous population between different vascular plexus in the same organ or across different organs. One functional aspect of this differentiation is because vascular endothelial cells serve as a major source of paracrine secretion signal to provide support for perivascular cells and tissue-resident cells in the corresponding organs. The combined application of high-throughput sequencing technologies as well as new models has led to the characterization and identification of heterogeneous endothelial populations at the transcriptomic level. The capillary endothelial cells used to be recognized as homogenous VEC cell population, but recent data suggests that the phenotypic and structural diversity of capillary VEC is highly dependent on the organs or tissue microenvironments in which they reside (Augustin and Koh 2017; Bondareva et al. 2022; Chen et al. 2024; Gomez-Salinero et al. 2025; Kalucka et al. 2020; Paik et al. 2020). Given that different organs have different needs, dissecting the functional heterogeneity of endothelial cells in healthy organs is a potential key to understand endothelial health and behavior, as well as a critical factor to determine how and why they become dysfunctional in disease.

This review focuses on our current understanding of vascular endothelial cell heterogeneity, including intra-organ heterogeneity and organ-specific VEC differences across various organs. It also highlights emerging tools, such as single-cell RNA sequencing (scRNA-seq) for dissecting VEC heterogeneity, and vascular organoids for investigating the functional significance of this heterogeneity. These advances aim to provide insights for future studies on how the proper differentiation of VEC into specific endothelial subtypes contributes to development, homeostasis, and disease states in organisms.

## The heterogeneity of endothelial cells

Vascular endothelial cell heterogeneity refers to the diverse variations in cellular morphology, gene expression, function, metabolism, and proliferative potential under both physiological and pathological conditions (Marziano et al. 2021; Teuwen et al. 2020). This heterogeneity occurs within a single organ (intra-organ heterogeneity) and across different organs (inter-organ heterogeneity) (Aird 2012). Intra-organ heterogeneity refers to the differences among vascular endothelial cells within the vascular tree, including arteries, veins, and capillaries. This heterogeneity is also evident in the differentiation of tip and stalk endothelial cells during sprouting angiogenesis, a key process by which VEC expand the vascular network. The inter-organ heterogeneity describes the variability of VEC to fulfill the unique physiological needs of different tissues (Potente and Mäkinen 2017). For instance, VEC in the blood–brain barrier (BBB) regulate selective permeability, while glomerular endothelial cells in the kidney influence filtration (Amersfoort et al. 2022; Wu et al. 2023). In recent years, the concept of VEC heterogeneity has gained significant attention, understanding this heterogeneity is vital for the development of targeted therapies for vascular diseases, as well as advancing regenerative medicine and tissue engineering.

### Intra-organ endothelial cell heterogeneity

#### Tip and stalk cell specification

**Fig. 2:**
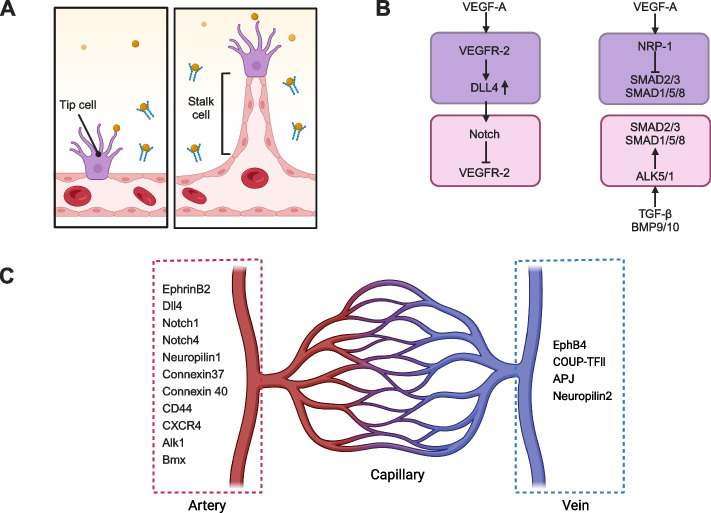
Intra-organ endothelial cell heterogeneity. **A** Tip-stalk endothelial cell heterogeneity which are induced by surrounding growth factor including VEGF to attract growth of blood vessel; **B** Key signal players during tip-stalk endothelial cell specification; **C** Arterial-venous endothelial cell heterogeneity and molecular markers for arterial or venous endothelial cells

During embryonic development, as tissues experience hypoxia, angiogenesis is triggered to vascularize these regions through the upregulation of VEGF-A, which promotes the sprouting of endothelial cells (Simons et al. 2016). The concept of tip endothelial cell and stalk endothelial cell specification is proposed during the investigation of sprouting angiogenesis (Fig. 2A) (Gerhardt et al. 2003; Makanya et al. 2009).

VEGF-A binds to its major receptor vascular endothelial growth factor receptor- 2 (VEGFR2) that is enriched in tip cells, supporting their survival and migration. Disruption of the VEGF-A/VEGFR2 signaling pathway severely impairs vascular development, often leading to early embryonic lethality (Apte et al. 2019). A key regulator of tip cell identity is the NOTCH ligand delta-like protein 4 (DLL4), which is preferentially expressed in tip cells and activated downstream of VEGF-A-VEGFR2 signaling (Pérez-Gutiérrez and Ferrara 2023). DLL4 interacts with NOTCH receptors on neighboring stalk cells, promoting VEGFR1 expression, a decoy receptor that sequesters VEGF-A, thus limiting VEGFR2 expression, inhibiting migration, and preserving the stalk cell phenotype (Blanco and Gerhardt 2013).

Other signaling pathways that contribute to tip-stalk cell specification include TGF-β and BMP9/10 signaling. Specifically, TGF-β and BMP9/10 activate TGF-β type-1 receptors, ALK5 and ALK1, respectively, in stalk cells, promoting stalk cell identity through SMAD2/3- and SMAD1/5/8-dependent signaling (Marziano et al. 2021). In contrast, VEGF-A-dependent activation of Neuropilin-1 (NRP-1) in tip cells inhibits SMAD signaling, thereby repressing stalk cell characteristics and reinforcing tip cell identity (Aspalter et al. 2015).

As the vascular plexus grows and matures, the complexity of maintaining tip and stalk cell identity increases. VEC branching from existing vessels is partially controlled by the interplay between BMP and NOTCH signaling (Akil et al. 2021). BMP signaling promotes VEC branching, while NOTCH signaling inhibits this process. In zebrafish, BMP6 and BMP2 promote VEC branching through a SMAD1/5/8-dependent mechanism, which is counterbalanced by NOTCH-driven activation of the inhibitory SMAD6 (Mouillesseaux et al. 2016). This balance carefully regulates vessel branching, ensuring proper vascular organization during development. The specification of tip and stalk endothelial cells is a critical cellular event during angiogenesis, drawing significant attention to the identification of key regulators of this process. Understanding the regulators of this intra-organ endothelial cell differentiation is essential for precisely promoting or inhibiting blood vessel growth.

#### Arterial-venous specification

As the embryo develops, VEC differentiate into arterial or venous phenotypes in response to a complex network of signals (Fig. 2B, C) (Marziano et al. 2021). Historically, arterial and venous VEC were believed to be identical, differing only in terms of blood flow speed. This concept changed when the laboratories of David Anderson and Rüdiger Klein independently identified EphrinB2 and EphB4 as molecular markers of arterial and venous endothelial cells, respectively (Adams et al. 1999; Gerety et al. 1999; Wang et al. 1998). The interaction between EphrinB2 and EphB4 helps to prevent the mixing of arterial and venous VEC, thereby preserving vessel identity during vascular development. VEC acquire their arterial or venous identity based on which signaling pathways are dominantly activated (Wiley et al. 2011). As blood flow begins, mechanical shear stress further solidifies the specification of arterial-venous specification.

Several signaling pathways contribute to early arterial-venous specification, including VEGF-A, WNT, and SHH (Corada et al. 2014). The fate of ECs depends on VEGF-A concentration: high levels promote arterial specification, while lower levels favor venous identity. In arterial-fated VEC, VEGF-A-VEGFR2 signaling activates NOTCH and ERK1/2 pathways, driving arterial gene expression (Pérez-Gutiérrez and Ferrara 2023). VEGF-A signaling also upregulates transcription factors SOX7, SOX17, and SOX18, which in turn increase NOTCH-1 expression, reinforcing arterial identity (Chiang et al. 2017). In zebrafish, BMP signaling enhances NOTCH-dependent arterial specification, where BMP4 activation of BMPER and TWSGI supports the upregulation of EphrinB2 through NOTCH signaling. Parallel VEGF-VEGFR2 signaling drives ERK1/2-mediated upregulation of arterial genes (Hong et al. 2006).

WNT signaling, downstream of VEGFR2, also induces arterial specification by promoting NOTCH expression. Baseline WNT/β-catenin signaling upregulates DLL4, further activating NOTCH in adjacent cells and establishing arterial fate. However, overactivation of this pathway can result in defects, such as the formation of arteriovenous shunts (Corada et al. 2010). SHH signaling is also involved, as SHH-VEGF-A-NOTCH signaling initiates arterial specification in zebrafish. Overexpression of SHH leads to ectopic arterial VEC formation, while inhibiting SHH results in the loss of arterial identity (Ehling et al. 2013).

The onset of blood flow through the developing vasculature plays a key role in reinforcing arterial identity (dela Paz and D’Amore 2009). In chick embryos, blood flow in yolk sac vessels triggers the expression of arterial-specific genes, including EphrinB2 (Broggini et al. 2020). Flow-mediated arterial specification is partly regulated by NOTCH, which upregulates the mechanosensitive gap junction protein Connexin 37 (Cx37) (Ehling et al. 2013). Cx37, in turn, activates the cell cycle inhibitor p27, leading to endothelial cell cycle arrest and the subsequent upregulation of arterial genes. The exact mechanism by which Cx37 controls p27 and cell cycle arrest is not fully understood and requires further investigation (Fang et al. 2017).

In contrast, venous VEC specification is promoted by inhibiting arterial specification. The venous-enriched transcription factor Nr2f2/COUP-TFII suppresses arterial identity by downregulating NOTCH signaling. Additionally, PI3K-AKT signaling, which opposes the ERK1/2 pathway, drives venous specification by promoting COUP-TFII expression. PI3K is also activated by VEGF-A, which can promote arterial specification, underscoring the complexity of the pathways involved in determining arterial-venous identity in response to VEGF-A signaling (Simons et al. 2016).

Arterial-venous specification is one of the most fundamental features of the vascular network. Understanding this process is not only crucial for vascular research but also highly relevant to arterial-venous vascular diseases, such as cerebral cavernous malformations.

### Inter-organ endothelial cell heterogeneity

The vasculature of each organ is adapted to meet the physiological function of that tissue. This organ-specific specialization is reflected in the structure, function, and molecular expression profiles of endothelial cells, as well as in the composition of perivascular cells and the surrounding extracellular matrix (Augustin and Koh 2017; Chi et al. 2003). Traditionally, endothelial cells are known to exhibit heterogeneous morphology across different organs, with three primary phenotypes: discontinuous (found in the bone marrow and liver), fenestrated (in the kidney and gastric and intestinal mucosa), and continuous (in the brain, heart, and lungs) (Gomez-Salinero et al. 2025; Potente and Mäkinen 2017). Electron microscopy is a powerful tool for observing the structural differences in these endothelial cells. Discontinuous endothelium has gaps between endothelial cells, forming large pores (100–200 nm in diameter) without diaphragms. This morphology facilitates the transport of large solutes, such as plasma proteins, and allows the free exchange of water and fluid. Blood flow around these sinusoidal endothelial cells slows to enable extensive material exchange. Fenestrated endothelium is typically found in areas with high filtration or frequent trans-endothelial transport. The fenestrae within the cells are accompanied by diaphragms that penetrate the endothelial cells. This type of endothelium is present in capillaries of exocrine and endocrine glands, the gastric and intestinal mucosa, the choroid plexus, glomeruli, and certain tubular regions of the kidney. In these areas, fluid exchange is rapid, and small solutes, such as small peptides, pass through the vessels into the interstitial tissue fluid. Continuous endothelium, found in tissues with lower exchange rates and tightly regulated barriers (e.g., the brain, heart, and lungs), features a continuous basement membrane without fenestrations, forming a tight barrier. Only water, small solutes, and lipophilic substances can diffuse into the surrounding tissue and interstitial fluid. This structure prevents the leakage of circulating cells and plasma proteins, maintaining strict permeability and blocking the entry of most large molecules and pathogens.

**Fig. 3:**
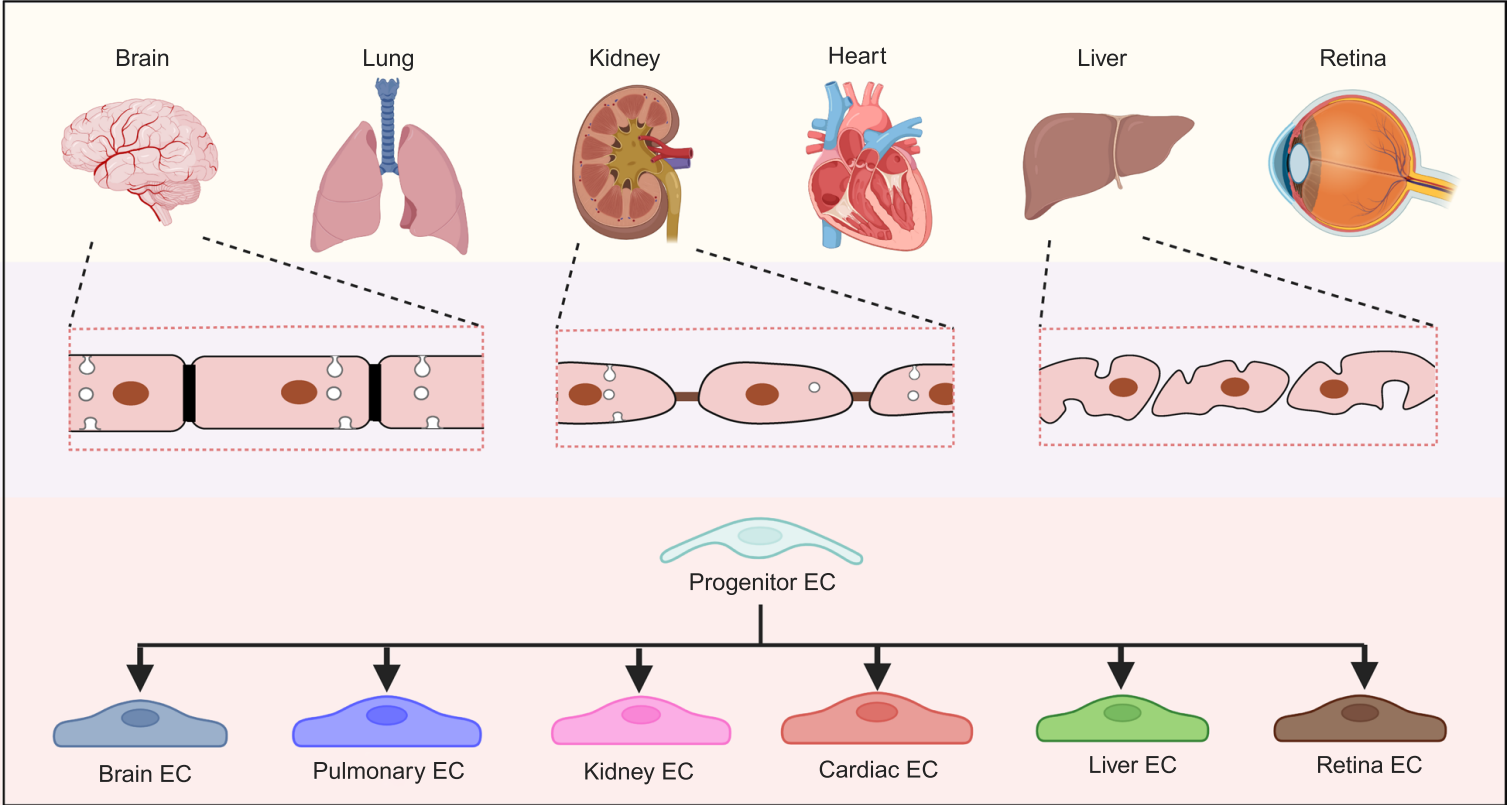
Inter-organ endothelial cell heterogeneity. The endothelial cells lining blood vessel has organ specificity. Brain vasculature is closely connected without any gaps. Kidney vasculature has gaps across endothelial cells to control permeability of material. Liver blood vessels specialize into the so-called sinusoidal blood vessels with. Therefore, it is essential to investigate how endothelial progenitor cells differentiate into organ-specific endothelial cells and investigate the functional diversity of endothelial cell heterogeneity across different organs, which needs intensive investigation in future

Understanding these unique characteristics is crucial for uncovering the mechanisms underlying various diseases and developing targeted therapeutic approaches. In every organ, the vasculature not only provides oxygen and nutrients but also plays a vital role in maintaining homeostasis, supporting immune function, and facilitating tissue repair (Potente and Mäkinen 2017). In this section, we will discuss the specialized vascular features in the brain, lung, heart, liver, kidney and retina, with a focus on the functional adaptations that allow these vessels to carry out their specific roles in each organ (Fig. 3).

#### Brain vasculature

In recent years, the vasculature of the central nervous system has been extensively investigated because of its pivotal role in human disease and aging (Vanlandewijck et al. 2018). During mouse embryonic development, the central nervous system (CNS) remains avascular until distinct brain compartments begin to form. Around embryonic day (E) 8.5, primitive vascular endothelial cells aggregate to form the perineural vascular plexus (PNVP) surrounding the central nervous system. These vascular endothelial cells subsequently differentiate into the arteries, capillaries, and veins of the meninges. Following this, blood vessels from the PNVP invade the parenchyma of central nervous system, extending along radial glial fibers towards the ventricles of brain. Wnt and other signaling pathways regulate the endothelial cell invasion into the parenchyma of central nervous system, reaching the subventricular zone of the brain. This process forms the intraneural vascular plexus (INVP), which undergoes further vascular remodeling (Quaegebeur et al. 2011; Walchli et al. 2023). At the cellular level, vascular endothelial cells recruit pericytes and associate with astrocyte endfeet, contributing to the formation of the neurovascular unit (Sweeney et al. 2018; Walchli et al. 2021). These data support the existence of CNS-specific angiogenesis mechanisms, particularly in vascular-dependent brain pathologies. In these conditions, the neurovascular unit is often dysregulated, angiogenic signaling pathways are aberrantly activated, and abnormal neovascularization patterns lead to leakage, tortuosity, and dysfunction (Iadecola 2017). The brain vasculature is a key component of the blood–brain barrier (BBB), where endothelial cells are tightly linked without large gaps (Fig. 3). This barrier function strictly regulates the passage of cells and nutrients into and out of the brain. In addition to this traditional knowledge, the brain capillaries have recently been revealed as a capillary bed with zonation features, in which endothelial cells exhibit a continuous transcriptional transition along an arterial-venous axis (Gurevich et al. 2021). This zonation reflects gradual changes in cellular phenotypes along an anatomical gradient and is first identified in the brain, with later confirmation in the heart, suggesting it is a broader feature of capillaries (Vanlandewijck et al. 2018). Interestingly, this zonation does not occur in mural cells adjacent to endothelial cells in the brain’s vasculature. Further studies have confirmed capillary zonation in human brains, early mouse and human embryos. Disruption in perivascular cell/endothelial cell communication is found to shift capillary zonation toward a venous identity without causing widespread BBB dysfunction (Armulik et al. 2010).

These molecular features of brain vasculature were linked to genetic variants associated with brain disease. For example, comparisons of gene expression in mice carrying Alzheimer’s disease-associated genetic variants, and altered expression in Alzheimer’s brain tissue, suggest that brain capillary endothelial cells may contribute to pathology by modulating BBB-related genes (Yang et al. 2022). The local damage to brain blood vessels leads to the loss of endothelial cells and a significant upregulation of inflammatory genes (Wang et al. 2024). Additionally, the absence of collagen IV, an endothelial cell-associated extracellular matrix protein, contributes to the breakdown of the blood–brain barrier. Inflammatory mediators, such as TNF-α, IL-6, and MCP-2, promote endothelial activation and accelerate the deposition of β-amyloid plaques, which release neurotoxic peptides that damage cortical neurons (Zhang et al. 2023b). Similar studies using human data have pointed to endothelial and mural cells as likely sites of disease onset in neurodegenerative conditions (Winkler et al. 2022; Yang et al. 2022; Zlokovic 2008). Therefore, it would be potentially interesting to investigate whether the zonation features of brain vasculature are associated with neurodegenerative disease.

#### Pulmonary vasculature

The pulmonary vasculature is unique in its dual role, serving both the circulatory and respiratory systems (Ellis et al. 2020). Its primary function is to transport deoxygenated blood from the heart to the lungs for oxygenation and then return oxygenated blood back to the heart for systemic circulation (Augustin and Koh 2017). This dual role requires a highly specialized vascular structure and function.

Pulmonary arteries and veins differ significantly from systemic vessels. Pulmonary arteries, with thinner walls and less smooth muscle, accommodate the low-pressure blood flow characteristic of the pulmonary circulation. Additionally, pulmonary endothelial cells are adapted to maintain low vascular resistance while ensuring efficient gas exchange across the alveolar-capillary barrier (Gillich et al. 2020).

Alveoli, the terminal airspaces in the lungs where gas exchange occurs, are surrounded by a dense network of capillaries (Augustin and Koh 2017). Recent studies have revealed significant heterogeneity within the alveolar vasculature. During late embryonic development, specialized capillaries known as Car4 + aerocyte capillaries (aCaps) emerge, interspersed with general capillaries (gCaps) (Gillich et al. 2020). CAR4 encodes carbonic anhydrase 4, which plays a role in the reversible hydration of carbon dioxide. While gCaps share markers with capillaries from other organs, aCaps express unique markers such as ICAM1 and EDNRB, which are specific to the lung. ICAM1 is known to regulate vascular permeability by interacting with corresponding receptors on leukocytes, thereby maintaining the barrier integrity of the lung (Bui et al. 2020). EDNRB acts as the receptor for endothelin-1, regulating the release of vasodilators to preserve the morphology of pulmonary blood vessels (Horinouchi et al. 2013). aCaps also have a distinct morphology, spreading over the thin alveolar type 1 epithelium where gas exchange occurs (Trimm and Red-Horse 2023). Their gene expression patterns and morphology suggest specialized roles in gas exchange.

The trajectory analyses of scRNA-seq and lineage-tracing data indicate that gCaps act as progenitors for aCaps during alveolar development and post-injury (Niethamer et al. 2020). VEGF signaling from the airway epithelium is crucial for the development and maintenance of aCaps, because VEGF deletion reduces aCap numbers without affecting gCaps. aCaps are essential for lung maturation, as their absence in mice leads to enlarged alveoli despite the presence of myofibroblasts (Ellis et al. 2020). This capillary heterogeneity has provided new insights into human lung pathology. For example, in lung adenocarcinoma and COVID-19, aCaps are either lost or co-express markers of both aCaps and gCaps, indicating disruptions in capillary identity (Wang et al. 2021). Additionally, aCaps mediate responses in conditions like bronchopulmonary dysplasia, highlighting their role in lung disease. In patients with advanced chronic obstructive pulmonary disease, pulmonary capillary endothelial cells show dramatic transcriptional changes in response to inflammation and stress, underscoring their importance during pulmonary diseases (Sauler et al. 2022).

#### Cardiac vasculature

The development of coronary blood vessels in the heart is primarily derived from the differentiation of endothelial cells from two distinct progenitor sources: the sinus venosus (an inlet vein) and the endocardium (which lines the heart chambers) (Li et al. 2019). These two cell populations localize to overlapping but distinct regions within the heart: sinus venosus-derived cells primarily form vessels in the outer ventricular walls, while endocardium-derived cells contribute mainly to the septum and inner ventricular walls (Red-Horse et al. 2010). Despite these spatial differences, endothelial cells from both progenitors begin to sprout around embryonic days 11 to 12.5 in mice, responding to different molecular signals—such as VEGFC-apelin receptor signaling for sinus venosus-derived cells or VEGFA signaling for endocardium-derived cells (Chen et al. 2014). Once these endothelial cells sprout into the developing heart, they undergo extensive remodeling, eventually forming coronary arteries, capillaries, and veins.

Postnatally, the heart undergoes rapid growth and remodeling, particularly in the trabecular myocardium, which lacks vessels during embryogenesis but later is full of coronary vascularization (Tan and Lewandowski 2020). Endocardium-derived vessels expand into this region after birth through lineage conversion, although this contribution is minimal in response to injury in later development (Tang et al. 2022). Differentiation of endocardial cells into coronary endothelial cells mainly occurs during early heart development, and the endocardium-derived vascular endothelial cells mature into arteries by postnatal day 7 (Tang et al. 2022). Postnatal angiogenic expansion of coronary vessels is driven by VEGFR3-expressing endothelial cells, with a key role for delta-like protein 4-NOTCH1 signaling in promoting vascularization of the newly compacted myocardium (Lu et al. 2021a).

Research has shown that coronary vessel growth can be enhanced under certain conditions, such as through VEGFB transduction or overexpression of VEGFR2 in adult mice. ScRNA-seq data have revealed that BMP2 expression is specific to the embryonic period when angiogenesis is active, and reactivation of BMP2 can induce endocardial angiogenesis in neonatal mouse models of myocardial infarction (Jiang et al. 2021).

Comparisons of capillary endothelial cell transcriptional states from lineage-traced hearts have shown that adult coronary endothelial cells do not retain molecular signatures of their progenitors, a phenomenon known as “convergent differentiation.” This has been observed in other systems but not in certain cell types like hematopoietic cells or tissue-resident macrophages, which maintain markers of their primed fate (Phansalkar et al. 2021). No functional differences have been observed between endothelial cells from different progenitors, as they exhibit similar proliferative responses to injury.

Recent data have revealed similarities in endothelial cell transcriptional states across species, validating the use of mouse models for studying many aspects of human coronary development. Functional experiments using induced pluripotent stem cell (iPSC) models have shown that MECOM, a histone-lysine N-methyltransferase, is critical for human artery endothelial cell differentiation (D’Amato et al. 2022; McCracken et al. 2022). Trajectory analyses have further identified a transitory cell population originating from the endocardium in both mice and humans, providing evidence that coronary vessels in humans may also arise from the endocardium.

#### Kidney vasculature

The kidney exhibits remarkable diversity and contains specialized endothelial cell populations (Potente and Mäkinen 2017). The renal microvasculature plays crucial roles, including regulating blood flow, facilitating filtration, modulating inflammation, and maintaining blood pressure. These functions are carried out by compartmentalized glomerular endothelial cells, which form the filtration barrier, while tubular and peritubular capillary endothelial cells are involved in fluid absorption and secretion (Muto et al. 2021).

Single-cell analyses of mouse models of kidney disease have shed light on the molecular differences between cortical, medullary, and glomerular endothelial cells and their unique responses to pathological conditions (Kim et al. 2024). For example, cortical endothelial cells express high levels of Igfbp3 (insulin-like growth factor binding protein 3) and Npr3 (natriuretic peptide receptor 3), while medullary endothelial cells express Igf1 (insulin-like growth factor 1) and Cd36 (platelet glycoprotein 4). Glomerular endothelial cells are enriched for genes such as Ehd3 (EH domain-containing protein 3), Cyp4b1 (cytochrome P450 4B1), and Tspan7 (tetraspanin 7) (Chung et al. 2020).

Under conditions of acute water deprivation, medullary endothelial cells undergo significant transcriptional changes, upregulating genes related to oxidative phosphorylation to support survival and enable urine concentration (Chung et al. 2020). In a mouse model of nephritis induced by nephrotoxic serum administration, disease progression was marked by endothelial cells shifting towards an activated state (Hackl et al. 2023). In contrast, in an eNOS null mouse model of diabetes mellitus, endothelial cell states closely resembled those in wild-type mice, suggesting different pathological responses between these conditions (Fu et al. 2019).

#### Liver vasculature

Liver endothelial cells play a crucial role in liver function, which includes receiving blood from both the hepatic artery and portal vein, scavenging macromolecules, and regulating immune responses (Augustin and Koh 2017). Recent studies have also explored how liver sinusoidal endothelial cells become dysregulated in conditions such as cirrhosis and hepatocarcinoma (Su et al. 2021).

A majority of studies map hepatocyte–endothelial cell pairs back to their organ locations, identifying molecular signatures for subpopulations of pericentral liver endothelial cells and characterizing endothelial cell zonation across different lobules of liver (Halpern et al. 2018). This spatial analysis showed strong concordance with proteomic data, indicating that liver zonation is driven by functional differences along the lobule axis, rather than a continuum of arterial-venous states as seen in the brain (Hildebrandt et al. 2021). Liver endothelial cell zonation is crucial for hepatocyte specialization, mediated by a TIE–WNT signaling axis. The transcription factor MAF is key to establishing zonation in embryonic liver endothelial progenitors and maintaining liver sinusoidal endothelial cell identity (Gómez-Salinero et al. 2022).

Liver sinusoidal endothelial cells, particularly in lobule zone 3, show specific susceptibilities to injury in disease states like cirrhosis, where zone 3 liver sinusoidal endothelial cells undergo capillarization—a loss of specialized transcriptional states. This process mirrors the loss of capillary identity seen in lung diseases and highlights the importance of endothelial cell zonation in liver health (Su et al. 2021). Factor VIII, secreted by liver sinusoidal endothelial cells, serves as a cofactor in the intrinsic coagulation pathway, and its deficiency results in hemophilia A (Do et al. 1999; Fomin et al. 2013). When liver sinusoidal endothelial cells are induced to produce Factor VIII and transplanted into an immunodeficient hemophilia A model, the bleeding phenotype is effectively corrected (Fama et al. 2021; Merlin et al. 2019). Exogenous retroviral injection studies reveal that Factor VIII is primarily expressed and produced by liver sinusoidal endothelial cells, with no immune suppression observed within one year after transplantatio (Fama et al. 2021; Merlin et al. 2019). This research highlights the potential of targeting specific paracrine factors in tissue-specific endothelial cells as a therapeutic approach for tissue-related diseases.

#### Retinal vasculature

The retinal vascular system is the most frequently used vascular research system in genetically modified mice and allows non-invasive visualization of the body’s vasculature, providing a unique and accessible window into the body’s vascular microcirculation in both healthy and diseased states (London et al. 2013). As a highly metabolically active tissue, the formation of the retinal vascular system is driven by multiple signaling pathways and cellular interactions. On one hand, endothelial cells proliferate to produce neovascularization in response to angiogenic stimuli, and dysregulation of angiogenesis causes nutrient deficiencies, metabolic imbalances, and neurological dysfunction. On the other hand, abnormal neovascularization and leakage due to endothelial abnormalities are common hallmarks of retinal degenerative diseases, including age-related macular degeneration (AMD)(Menon et al. 2019), diabetic retinopathy (DR)(Yao et al. 2024), retinopathy of prematurity (Ramshekar et al. 2022), retinopathy of pigmentation (Kubota et al. 2020), and other retinal degenerative diseases (Kaur and Singh, 2023). Therefore, a precise regulation of retina vascularization is essential to maintain normal retinal function.

The metabolic supply of the retina depends on the choroidal vasculature and the endo-retinal vasculature, both of which undergo dramatic changes and reorganization during development (Campochiaro 2021). The intraretinal vascular system emanates from the central retinal artery and gives rise to three parallel and interconnected vascular networks: the primary plexuses located in the nerve fiber layer, and the deep and intermediate plexuses, arranged on either side of the inner nuclear layer. In turn, the choroidal vasculature is supported by collagenous and elastic connective tissue containing melanocytes, fibroblasts, and resident immunoreactive cells (Burns et al. 2021). Since the sequential development of each plexus depends on different underlying mechanisms and is designed for dynamic structural changes, the endothelium of the different regions exhibits unique characteristics.

The origin of plexus network formation is the vascular sprouting extension (Mleynek and Li 2013). Tip cells direct the direction of growth, sensing the concentration gradient of VEGF and other growth factors and suppressing the tip phenotype of neighboring endothelial cells through the Notch signaling pathway (Chen et al. 2019a). In contrast, cells at the base of the sprout proliferate and form vascular lumens that maintain bud extension and perfusion (Chen et al. 2019a). Thus, individual cells can dynamically flip between tip and stalk phenotypes. Mouse retinal vascularization undergoes three main periods, starting from 1 day after birth when sprouts from the lateral side of the optic stalk migrate from the retinal surface to the periphery (P0-P7), followed by the P7-P21 stage when sprouting vessels detach from the superficial layer and extend into the deep outer plexiform layer, and then form an intermediate vascular plexus in the inner plexiform layer (Selvam et al. 2018). Microarray analysis of retinal and choroidal samples revealed that the most pronounced endothelial differences were detected between retinal and choroidal endothelial cells (Smith et al. 2007). This finding clearly demonstrates the existence of vascular endothelial diversity even within the eye and provides evidence of a unique molecular phenotype of retinal endothelial cells. By characterizing capillary-specialized endothelial cells, a tip cell type was identified that controls deep retinal vascularization (diving tip cell), which shifts to an S-like phenotype in the postnatal retina in response to endothelial-specific deletion of TGF-β receptor I (ALK5), inhibiting neuro-retinal vascularization (Zarkada et al. 2021). In DR and AMD, the retina exhibits marked metabolic imbalances and vascular changes, including thickening of the basement membrane, apoptosis of pericytes and endothelial cells, and diffuse increases in vascular permeability (Antonetti et al. 2021; Lin et al. 2024; Singh 2022).

Future explorations of endothelial heterogeneity in the retina need to consider how endothelial heterogeneity directs itself into a pathological state, and how these state results in disease progression.

### Endothelial cell heterogeneity in physiological and pathological conditions

In addition to the intra-organ and inter-organ heterogeneity, endothelial cells also display heterogeneity across disease conditions, aging as well as different genders. We briefly discuss the emerging understanding of endothelial cell heterogeneity in this aspect.

#### Endothelial cells in homeostasis and disease

Pathological conditions can alter endothelial cell profiles compared to healthy tissues in the following condition:

1. Changes in Endothelial Cell Proportions: In diseases such as Alzheimer’s disease, there is an increased presence of endothelial cells in the brain (Lau et al. 2020), whereas, in idiopathic pulmonary fibrosis, bronchial endothelial cells are more abundant, while other subtypes like arterial, venous, and capillary endothelial cells decrease in proportion across the pulmonary system (Broggini et al. 2020).
2. Specific Endothelial Subpopulations in Disease: In atherosclerotic plaques, endothelial cells expressing pro-inflammatory and pro-atherosclerotic genes are “activated,” contributing to disease progression (Depuydt et al. 2020).
3. Disease-Mediated Transcriptional Changes: Disease conditions can induce new endothelial subtypes. For example, genes associated with Class II major histocompatibility complex (MHC-II) are downregulated in pulmonary tumor endothelial cells (Goveia et al. 2020).
4. Endothelial-Cell Interactions in Disease: In atherosclerotic plaques, enhanced interactions between myeloid cells and endothelial cells—mediated by platelet-derived growth factor (PDGF) and PDGF receptor β (PDGFRB) signaling—promote angiogenesis (Depuydt et al. 2020).
5. Endothelial Transdifferentiation: Endothelial cells can transdifferentiate into other cell types by losing endothelial markers and acquiring those of other cells. In mice, mesenchymal gene activation is observed 7 days post-myocardial infarction, suggesting endothelial-mesenchymal transition in response to disease (Tombor et al. 2021).

#### Heterogeneity in young and aging endothelial cells

The young and aging endothelial cells display dramatic differences in several aspects:

1. Vascular Wall Structural Changes: Aging results in structural changes in the vascular wall, including increased stiffness of the middle and inner layers, decreased elasticity, endothelial dysfunction, impaired angiogenesis, and defects in vascular repair (Chen et al. 2016).
2. Inflammatory Responses and Oxidative Stress: Vascular aging is associated with oxidative stress, mitochondrial dysfunction, impaired resistance to molecular stressors, and chronic low-grade inflammation (Liu et al. 2023; Ungvari et al. 2018).
3. Gene Expression and Molecular Marker Changes: Aging reduces the proportion of capillary endothelial cells in brain tissue. Differential gene expression analysis reveals a marked upregulation of genes involved in inflammatory and immune-related signaling pathways (Ximerakis et al. 2019).
4. DNA Repair and Cell Interactions: DNA repair-related pathways are downregulated in aging endothelial cells, and the interaction between endothelial cells and fibroblasts is lost. This is accompanied by abnormal PTX3 secretion in fibroblasts, leading to sclerosis and vascular atrophy in aging skin (Ichijo et al. 2022).

#### Heterogeneity of endothelial cells in male and female mice

Recent data reports the endothelial cell heterogeneity in different genders because of the following reasons:

1. Sex-Specific Endothelial Subsets: Specific endothelial subsets exist in organs such as the aorta, brain, and lungs in both male and female mice (Paik et al. 2020). Differential gene expression in these subsets is influenced by sex. For instance, LARS2 is highly enriched in endothelial cells from male brain, ventricles, liver, and lung tissue, with its variant linked to multi-organ dysfunction and sex-specific phenotypic differences (Riley et al. 2020).
2. Sex Hormones and Endothelial Function: Sex hormones significantly impact vascular function and endothelial cell health. Estrogen triggers signaling through ERα, ERβ, or GPER-1, activating endothelial nitric oxide synthase (eNOS) to produce nitric oxide (NO), which regulates flow-mediated vasodilation (Davezac et al. 2021). Estrogen also modulates vasoconstrictors such as endothelin (ET-1) and prostacyclin (PGI2), influencing vascular tone (Marcantoni et al. 2012). Estrogen can either inhibit or activate reactive oxygen species (ROS) depending on its concentration.
3. Testosterone’s Effect on Endothelial Cells: In males, testosterone promotes the proliferation of endothelial progenitor cells by binding to androgen receptors. At supraphysiological concentrations, testosterone also stimulates the proliferation and migration of endothelial cells (Gaba et al. 2018; Marcantoni et al. 2012).
4. Impact of Menopause on Cardiovascular Health: Postmenopausal women experience an increased risk of cardiovascular diseases, highlighting the role of female hormones—particularly estrogen and progestogens—in maintaining endothelial health and influencing cardiovascular disease development (Fadini et al. 2008).
5. Female Endothelial Cell Characteristics in Atherosclerosis: Female endothelial cells exhibit heightened levels of ROS, mitochondrial damage, apoptosis, and reduced angiogenic potential. Atherosclerotic lesions in females show greater accumulation of endothelial oxidative stress products and endothelial-to-mesenchymal transition (Robert 2023; Shin et al. 2024).

## Emerging new tools to investigate endothelial cell heterogeneity

### Applications of scRNA-seq in endothelial research

**Fig. 4:**
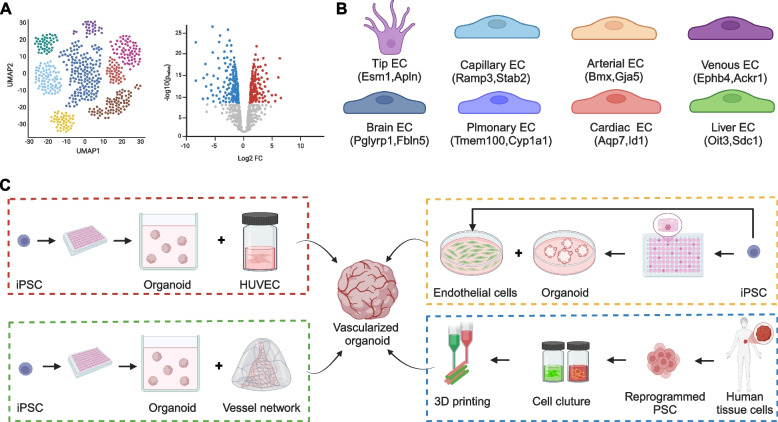
ScRNA-seq and organoids are emerging tools to investigate endothelial cell heterogeneity. **A** ScRNA-seq facilitate identification of new endothelial cell subpopulation and differentially-expressed genes. **B** Some molecular markers of different subtypes of heterogenous endothelial cells, which are revealed by scRNA-seq. **C** Different types of method to generate vascular organoids

In the previous sections, we describe the intra-organ and inter-organ endothelial cell heterogeneity. High-throughput sequencing technologies have greatly advanced our understanding of VEC heterogeneity. Several laboratories pioneered the use of microarray technology to compare endothelial cells across organs at a high-throughput level (Chi et al. 2003; Nolan et al. 2013). However, the limitation of this method prevents data comparison in different experiments. For example, microarrays are prone to high levels of noise and detection limitations, making it challenging to accurately capture smaller gene differences. Furthermore, microarray data report relative gene expression changes across different groups within a single batch of samples. However, datasets from one microarray batch cannot be directly compared to those from another batch (Bolon-Canedo et al. 2019; Richard et al. 2014). The rapid development of scRNA-seq has become an invaluable tool to map the endothelial cell landscape across different tissues and disease states (Fig. 4A, B). By analyzing transcriptional profiles at the single-cell level, scRNA-seq enables the identification of disease-associated endothelial cell subsets and the signaling pathways that drive the pathological conditions in health and disease across virtually all stages of life (e.g., development, adulthood, aging), as summarized in Table 1.

**Table 1:** Single-cell RNA sequencing summary

Organ	Species	Development	Homeostasis	Disease	ScRNA-seq method	References
Multiple organs	Mouse		✓		NextSeq500	Yao et al. 2018
	Mouse		✓		Microwell-Seq	Han et al. 2018
	Mouse		✓		Smart-Seq2	He et al. 2018
	Mouse		✓		10 × Genomics Chromium	Kalucka et al. 2020
	Mouse		✓	✓	10 × Genomics Chromium	Rohlenova et al. 2020
	Mouse		✓		10 × Genomics Chromium	Sabbagh et al. 2018
	Human		✓		10 × Genomics Chromium	Barnett et al. 2024
Skin	Human		✓		10 × Genomics Chromium	Li et al. 2021
Endothelial cells	Human				10 × Genomics Chromium	Liu et al. 2021
	Human				10 × Genomics Chromium	Paik et al. 2020
Lung	Mouse, human			✓	10 × Genomics Chromium	Schupp et al. 2021
	Mouse, human			✓	10 × Genomics Chromium	Goveia et al. 2020
Angiocarpy	Mouse		✓		Ovation RNA-Seq V2	Lother et al. 2018
	Mouse	✓			Smart-seq2	Su et al. 2018
	Mouse	✓	✓	✓	sNucDrop-seq	Hu et al. 2018
	Mouse			✓	10 × Genomics Chromium	Li et al. 2019
	Mouse			✓	10X Genomics GemCode	Cochain et al. 2018
	Mouse			✓	10 × Genomics Chromium	Wang et al. 2020
	Mouse			✓	10 × Genomics Chromium	Tombor et al. 2021
	Human		✓	✓	SORT-seq	Gladka et al. 2018
Liver	Mouse		✓	✓	NextSeq550	Inverso et al. 2021
	Human		✓		Self-developed	Halpern et al. 2018
	Human			✓	10 × Genomics Chromium	Zhang et al. 2020
Eye	Mouse			✓	BD Rhapsody scRNA -seq platform	Xia et al. 2023
	Mouse	✓			10 × Genomics Chromium	Zarkada et al. 2021
	Mouse		✓		10 × Genomics Chromium	Lehmann et al. 2020
	Human		✓	✓	10 × Genomics Chromium	Voigt et al. 2019
Kidney	Mouse	✓	✓		Illumina Bio-Rad	Barry et al. 2019
	Mouse		✓	✓	10 × Genomics Chromium	Dumas et al. 2020
	Human			✓	10 × Genomics Chromium	Young et al. 2018
Brain	Mouse, human		✓	✓	10 × Genomics Chromium	Garcia et al. 2022
	Human			✓	10 × Genomics Chromium	Yang et al. 2022
Carotid artery	Mouse			✓	10 × Genomics Chromium	Andueza et al. 2020

#### Use scRNA-seq to elucidate endothelial heterogeneity during development, homeostasis, aging and disease

As discussed above, scRNA-seq has revolutionized the understanding of endothelial cell heterogeneity, offering insights that were previously unattainable with traditional bulk RNA sequencing (Kalucka et al. 2020). While bulk sequencing provides an average gene expression profile from a mixed population of cells, scRNA-seq allows for the examination of individual endothelial cells, uncovering rare cell populations, novel cell types, and dynamic transcriptional changes during development, homeostasis, and disease progression (Griffiths et al. 2018). This high-resolution view has made scRNA-seq essential for unraveling the complexities of endothelial biology, particularly in studying the diversity of endothelial cells in different vascular beds and their responses to physiological and pathological stimuli (Augustin and Koh 2017). One of the key contributions of scRNA-seq has been its revelation of the diversity within the endothelial cell population. Previously considered a relatively uniform cell type, endothelial cells are now understood to exhibit significant variation in gene expression, functional characteristics, and responses to stimuli. This heterogeneity is seen not only between different organs but also within the same organ, where endothelial cells express unique molecular signatures depending on their location within the vascular tree (Depuydt et al. 2020; Hennigs et al. 2021).

For example, scRNA-seq has identified specialized endothelial cell populations in the brain that are responsible for maintaining the blood–brain barrier (BBB) (Pellegrini et al. 2020). These endothelial cells are marked by high expression of tight junction proteins and transporters critical for BBB integrity, setting them apart from endothelial cells in other tissues (Min et al. 2024). ScRNA-seq facilitated identification of aCaps and gCaps pulmonary endothelial cells forming the alveolar capillaries complex ‘Swiss-cheese’-like morphologies to surround each alveolus (Gillich et al. 2020; Schupp et al. 2021). Similarly, in the liver, scRNA-seq has identified sinusoidal endothelial cells with distinct transcriptional profiles that reflect their specialized roles in filtering blood and facilitating nutrient exchange, compared to systemic endothelial cells (Li et al. 2023; Winkler et al. 2021). In the kidney, scRNA-seq has revealed different endothelial populations in the glomerulus and peritubular capillaries, each with specific gene expression patterns that align with their roles in filtration and reabsorption (Chung et al. 2020; Liao et al. 2020). These findings underscore the importance of endothelial specialization in organ function and disease, providing new insights into the pathogenesis of disorders (Lake et al. 2023; Ramachandran et al. 2019; Wu et al. 2022; Yang et al. 2021).

ScRNA-seq has also been pivotal in mapping the dynamic changes that endothelial cells undergo during development and steady-state maintenance (Rohlenova et al. 2020). During embryonic development, endothelial cells transit from mesodermal progenitors to form primitive vascular networks through vasculogenesis, followed by angiogenesis to refine these networks (Dejana et al. 2017). ScRNA-seq captures these transitions at single-cell resolution, enabling the identification of key transcription factors and signaling pathways that drive endothelial cell specification and maturation (Becker et al. 2023; Gómez-Salinero et al. 2022). In adult tissues, scRNA-seq has been used to study how endothelial cells respond to various physiological challenges, such as hypoxia, shear stress, and inflammation. For instance, in models of ischemic injury, scRNA-seq has revealed that endothelial cells upregulate genes involved in angiogenesis and tissue repair, including components of the VEGF and Notch signaling pathways, reflecting their role in promoting neovascularization and restoring blood flow (Räsänen et al. 2021).

In vascular aging research, scRNA-seq has revealed transcriptional changes in endothelial cells as they age, identifying endothelial senescence as a key contributor to age-related vascular diseases (Trimm and Red-Horse 2023). During aging, scRNA-seq studies have shown increased expression of senescence markers and pro-inflammatory cytokines in endothelial cells, contributing to vascular dysfunction and age-related diseases (Ma et al. 2021). Aged endothelial cells show increased expression of genes involved in oxidative stress and inflammation, leading to the decline in vascular function observed in elderly individuals (Guo et al. 2018). These findings have the potential to guide the development of early detection biomarkers and therapeutic interventions aimed at mitigating age-related vascular decline.

In disease contexts, scRNA-seq has provided critical insights into endothelial cell heterogeneity within tumors, a key area of interest due to the role of tumor vasculature in cancer progression and resistance to therapy (Davidson et al. 2020). Tumor endothelial cells (TEC) exhibit distinct gene expression profiles compared to normal endothelial cells, with upregulation of genes involved in angiogenesis, immune evasion, and extracellular matrix remodeling. These findings have helped identify new therapeutic targets for anti-angiogenic therapies and improve our understanding of how tumors develop resistance to these treatments (Zeng et al. 2023). For example, scRNA-seq has uncovered TEC populations with high expression of angiopoietin-2 (Ang-2), a marker associated with poor prognosis in cancer patients. Understanding TEC heterogeneity has opened new avenues for developing more effective anti-angiogenic therapies and predicting patient responses to treatment (Goveia et al. 2020).

In summary, scRNA-seq has transformed vascular research by providing detailed insights into endothelial cell heterogeneity and dynamic changes during development, homeostasis, aging and disease conditions. These insights offer new avenues for understanding vascular biology and developing targeted therapies for vascular-related diseases and cancer.

#### Future direction and challenges using scRNA-seq in vascular biology

Despite its transformative impact, scRNA-seq technology still faces several challenges in endothelial research. One of the main limitations is the difficulty of capturing low-abundance endothelial cells, especially in tissues with high cellular diversity (Tan et al. 2023). The tissue dissociation process required for single-cell isolation can result in the loss of fragile endothelial cells or introduce artifacts that may affect the accuracy of the transcriptional profiles. Additionally, the high cost and technical complexity of scRNA-seq remain barriers for many research laboratories (Kharchenko 2021).

To overcome these challenges, future developments in scRNA-seq will likely focus on improving sensitivity, accuracy, and accessibility. Advances in microfluidics and single-cell capture techniques may enhance the recovery of low-abundance endothelial cells, while new computational methods could mitigate technical artifacts and provide better resolution of rare cell populations (Trimm and Red-Horse 2023). Reducing the cost and complexity of scRNA-seq technology will also help broaden its use in various research settings.

In addition to characterizing and identifying VEC phenotypes at the transcriptional level in both healthy and diseased states, a series of methods has been developed to perform multi-omics analyses of individual cells. These methods enable the parallel measurement of intercellular heterogeneity in the genome, epigenome, transcriptome, and proteome. The joint analysis of these measurements in multidimensional VEC has the potential to reveal the regulatory and functional mechanisms underlying cellular behavior in healthy development and disease. Integrating scRNA-seq data with other omics technologies, such as single nuclei RNA sequencing (snRNA-seq), single-cell ATAC-seq, spatial transcriptomics, and proteomics, will offer a more comprehensive understanding of endothelial cell heterogeneity (Wan et al. 2023).

Due to the challenges in dissociating tissue samples while maintaining their viability, extracting reliable information from clinical frozen samples, freshly collected tumor specimens, and delicate tissues has made the application of scRNA-seq difficult (Slyper et al. 2020). The introduction of single nuclei RNA sequencing (snRNA-seq) has offered a solution to these problems. Unlike scRNA-seq, which analyzes whole cells, snRNA-seq uses distinct dissociation methods, detecting individual nuclei after the cell membrane ruptures, thereby minimizing cell coverage bias (Andrews et al. 2022; Guo et al. 2023; Sun et al. 2020). Not constrained by small-scale microplates, snRNA-seq has successfully uncovered the heterogeneous subpopulations of endothelial cells after birth and their functional remodeling in pediatric cardiomyopathy (Habib et al. 2017; Hu et al. 2018). In snRNA-seq of the frontal–temporal dementia (FTD) brain cortex, genes associated with blood–brain barrier dysfunction were notably enriched in endothelial cells, contributing to abnormal vascular proliferation (Gerrits et al. 2022).

Combining scRNA-seq with spatial transcriptomics allows researchers to map endothelial cells’ spatial distribution within tissues, linking their transcriptional profiles to their anatomical locations and interactions with other cell types. This approach is particularly valuable in studying complex tissues, such as the brain, where endothelial cells play key roles in maintaining the blood–brain barrier and supporting neural function (Yao et al. 2021). Using scATAC-seq, epigenomic and transcriptomic analyses of the endothelium can be performed in the same sample (Danese et al. 2021). Functionally specific transcriptional profiles and chromatin accessibility of the proximal tubule and posterior ascending branch have predicted transcription factors characterizing the cell type specificity of the proximal tubule and thick ascending branch, as demonstrated by concatenated sequencing of adult kidney snRNA and snATAC-seq (Muto et al. 2021). By analyzing the interplay between chromatin accessibility and cis-regulatory elements, it has been clarified that perturbed fluid flow regulates gene reprogramming in endothelial cells, inducing atherosclerosis at both transcriptional and epigenetic levels (Andueza et al. 2020). This method has paved the way for other technologies that profile both the epigenome and transcriptome in single nuclei, such as Sci-CAR-seq (Cao et al. 2018), the single-nucleus chromatin accessibility and mRNA expression sequencing (SNARE-seq) (Chen et al. 2019a) and 10 × Multiome (Baysoy et al. 2023).

Cellular indexing of transcriptomes and epitopes by sequencing (CITE-seq) labels single cells using antibody conjugates bound to biotinylated DNA barcodes (Stoeckius et al. 2017). Despite significant advances in experimental methods for analyzing multiple histological patterns simultaneously, a major obstacle in the field remains the inherent complexity of integrating multi-omics data (Baysoy et al. 2023). The rapid development of spatial transcriptomics has addressed the gap of in situ information. When combined with spatial transcriptomics, single-cell expression data from the kidney enable clear identification and molecular characterization of anatomically defined cells, revealing cell type-specific gene expression changes in disease states (Abedini et al. 2024). Additionally, spatial DNA-MERFISH has provided complementary views of cells in spatial segments of tissues, offering deeper insights into cellular and emerging tissue properties (Liu et al. 2022). Integrated multi-modal analysis of single-cell transcriptomics and MERFISH-based presentation of spatial information reveals that specific combinations of pairs of spatial cell populations reveal different signaling pathways while being critical for the maintenance of cardiac function (Farah et al. 2024; Zhang et al. 2023a). While genetic characterization of heterogeneous cells using transcriptomics is powerful, it does not fully capture cellular processes and nuclear functions, which are predominantly governed by proteins. cellular indexing of the transcriptome and epitopes (CITE-seq) addresses this gap by incorporating protein characterization into transcriptome assays (Stoeckius et al. 2017). The emergence of SCoPE-MS and SCoPE2 has further enhanced the ability to comprehensively characterize cellular proteins with antibody-independent advantages (Budnik et al. 2018; Petelski et al. 2021).

Lineage tracing combined with scRNA-seq provides valuable insights into the lineage and gene expression of endothelial cells. For example, Hou et al. used this approach to explore endothelial heterogeneity and lineage relationships during early vascular development. Their study revealed that endothelial cells with venous characteristics in the embryonic vascular plexus rapidly acquire arterial features, a process that occurs concurrently with cell expansion and precedes the development of major organs in the embryo (Hou et al. 2022). In addition to transcriptional analysis, the emerging use of ChIP-seq has significantly advanced the study of chromatin-binding protein distribution across the genome. This technique effectively uncovers the epigenetic heterogeneity and cis-regulatory mechanisms of specific endothelial cell subpopulations. By integrating chromatin-binding data with transcriptional information, a more comprehensive understanding of gene expression in endothelial cells can be achieved (Wang et al. 2019). Furthermore, endothelial-specific translational ribosome affinity purification combined with high-throughput RNA sequencing (TRAP-seq) minimizes the transient changes in expression profiles often caused by cell sorting or tissue digestion. This method successfully captures low-abundance transcripts, overcoming the limitations of traditional techniques and enhancing the analysis of endothelial cell-specific expression in both physiological and pathological conditions (Cleuren et al. 2019; Lu et al. 2024). Through proteomic analysis, key regulatory molecules involved in endocytosis and receptor cycling were identified, with ARF6 emerging as a candidate molecule mediating changes in endothelial cell vesicular transport. Combining transcriptomic and proteomic data offers a deeper understanding of endothelial cell heterogeneity (Todorov-Volgyi et al. 2024).

Introducing these emerging technologies into the study of VEC heterogeneity offers novel approaches for understanding and characterizing the heterogeneous endothelium. Despite these advancements, challenges remain. In single-cell histology-linked technologies, while capable of resolving intercellular interactions from in situ information, are limited by low resolution, restricted single-cell coverage, and incremental program costs. Additionally, the requirement for cellular activity during sequencing hampers the capture of complete DNA and RNA from clinical sample (Sakamoto et al. 2020). Similarly, the low resolution and small capture area of spatial association technologies hinder the ability to fully identify and characterize individual cell and intact tissue transcriptional patterns. Future investigations should focus on tailoring these technologies to better serve the study of VEC.

### Applications of organoid in endothelial research

#### Current approaches to generate vascular organoids

Organoids are promising research model for constructing organ morphology and enabling personalized precision therapy in vitro. These microstructures reproduce the distinct morphologies and key features of organs through directed differentiation in vitro (Liu et al. 2021). The vascular system not only ensures a stable supply of nutrients and the removal of metabolic waste, but also regulates tissue homeostasis, regeneration and organ function (Pi et al. 2018). Due to the low diffusivity of oxygen and nutrients, constructing and perfusing vascular networks within organoids is essential to ensure adequate oxygen and nutrient distribution in larger organoids. Notably, most knowledge of endothelial cell heterogeneity has been derived from studies using genetically modified animals or two-dimensional (2D) cell culture systems. There is an urgent need for a system to investigate the function of human endothelial cells in three-dimensional (3D) organ development and disease progression. The vascular organoids are the most promising approach to fulfil this gap at present.

Current approaches to achieve vascularization of organoids include (Fig. 4C): (1) co-culturing organoids with endothelial cell lines; (2) co-culturing organoids with vascular organoids; (3) co-differentiating stem cells into organ and vascular cells; (4) utilizing 3D printing technique to create perusable organoids for vascularization (Table 2) (Frenz-Wiessner et al. 2024; Naderi-Meshkin et al. 2023; Salewskij and Penninger 2023; Wimmer et al. 2019). Induction of endothelial cell differentiation from hPSC is a key step in most of these approaches. ETS variant transcription factor 2 (ETV2) is a master regulator widely used in the reprogramming process from hPSC to endothelial cells (Elcheva et al. 2014; Liu et al. 2015; Ng et al. 2021; Shi et al. 2014). The transient introduction of ETV2 is a common approach during the differentiation of hPSC into endothelial cells (Ginsberg et al. 2015; Lu et al. 2021b; Morita et al. 2015). By modifying ETV2 at the mesodermal stage of differentiation, hPSC can efficiently and consistently differentiate into endothelial cells with stable, perfusable functions in vivo (Wang et al. 2020). Additionally, ectopic expression of ETV2 in human embryonic stem cells supports the formation and functional maturation of human-derived cortical organoids (Cakir et al. 2019; Lu et al. 2021b). Furthermore, exogenous ETV2 expression can directly convert fully differentiated non-VEC into functional VEC (Lee et al. 2017; Morita et al. 2015). By inhibiting venous-specific signaling and inducing arterial endothelial signaling pathways, hPSC can generate venous endothelial cells and arterial VEC with distinct transcriptional and functional characteristics (Ang et al. 2022). Recent studies demonstrate the ability to generate arterial-like and venous-like endothelial cells from pluripotent stem cells, which can precisely mimic disease-related mutations (Pan et al. 2025). The proper differentiation of endothelial progenitor cells into arterial VEC is crucial for vascular development and disease progression. For instance, Zhang et al. utilized the EFNB2-tdTomato/EPHB4-EGFP reporter system to generate functional arterial VEC from hPSC, showing that functional arterial VEC improved survival in a mouse myocardial infarction model (Zhang et al. 2017). Additionally, venous-derived VEC from hPSC can effectively supplement liver sinusoidal endothelial cells during liver transplantation (Gage et al. 2020). These studies underscore the importance and feasibility of generating human VEC from hPSC for potential clinical applications in disease research.

**Table 2:** Organoid methods and applications

Organ	**Build method**	**Cell source**	**Sequencing**	**Application**	**References**
Blood vessel	Millifluidic chips	hiPSC	No	A model to vascularize diverse biological 3D tissues	Quintard et al. 2024
	3D chips	HUVEC	No	Developing vascularized organ on chip and human disease models	Kim et al. 2013
	Hydrogel bio-print	HUVEC	No	A technique for vascularization of hydrogel constructs	Bertassoni et al. 2014
	Millifluidic chips	HLFs, HUVEC	No	A long-term tissue culture in vitro	Nashimoto et al. 2017
	Microfluidic chambers	HUVEC	RNA-seq	Metabolic, immunological and physiochemical studies and screens	Palikuqi et al. 2020
	Enveloped by basement membrane	hPSC	No	Identifying the regulators of diabetic vasculopathy	Wimmer et al. 2019
Kidney	Differentiation culture	hPSC	scRNA-seq	Personalized medicine validation	Low et al. 2019
	3D print, Millifluidic chips	hPSC	No	Organ Culture Techniques; Organoids growth development	Homan et al. 2019
Brain	Transplantation	hiPSC	No	Brain organovascularization therapy	Pham et al. 2018
	Spinning culture	hESC	scRNA-seq	Transplantation	Cakir et al. 2019
	Cultured in shaker suspension	hiPSC	No	In vivo imaging; immunohistochemical analysis	Wilson et al. 2022
	Orbital shaker, spinning flask	hESC	No	Transplantation; diagnostic imaging	Mansour et al. 2018b
	Matrigel embedding	hESC	RNA seq	Treatment	Ham et al. 2020
	Transplantation	hPSC	No	Overcomes some limitations of in vitro systems	Lancaster 2018
	Shaker incubator	hESC	scRNA-seq	Modeling interactions between the neuronal and non-neuronal components in vitro	Sun et al. 2022
	Co-culture	hESC or hiPSC, HUVEC	scRNA-seq	Studying human cortical development and explore brain disease pathology	Shi et al. 2020
Cancer	Millifluidic chips	Patient tumor	No	Drug screening	Ko et al. 2024
	3D microvessel model	Breast cancer, HUVEC	No	Tumor-vascular dynamics	Silvestri et al. 2020
Heart	3D print	Reprogrammed PSC	No	Engineering personalized tissues and organs; drug screening	Noor et al. 2019
Liver	Transplantation	iPSC-Hes	No	Treatments for patients	Takebe et al. 2013
Bone marrow	Transplantation	hiPSC	scRNA-seq	In vitro model to study hematopoietic development and BM diseases	Frenz-Wiessner et al. 2024

#### Tissue vascularization in organoid models

The previous-mentioned approaches are utilized to generate organ-specific organoids, which could be useful to investigate endothelial cell heterogeneity in 3D in vitro model, which are summarized below.

##### Brain

The differentiation of human pluripotent stem cells into cerebellum-like structures, known as brain organoids, offers an unprecedented opportunity to model human brain development and disease. Co-culturing hESC or hiPSC-derived lineage organoids with human umbilical vein endothelial cells (HUVEC) to generate vascularized organoids and implanted them into mice, revealed that these transplanted organoids survived and integrated into the host cortical tissue (Mansour et al. 2018a; Shi et al. 2020). Furthermore, Brain organoids derived from hESC were fused with vascularized organoids and cultured into vascularized brain organoids. These exhibited more complex functional barrier architectures and microglia populations capable of responding to immune stimuli, thereby mimicking the interactions between the vascular system and neuronal components in vitro (Sun et al. 2022). Beyond classical induced organoids, 3D printing offers immense potential for constructing stable, vascularized brain organoid structure. The development of perfusable neurovascularized organoids using tissue-chip-based microfluidic systems also holds significant promise for the advancing complex neurovascular units (Salmon et al. 2022). However, while perfusion has been partially achieved in existing vascularized organoids, the lack of immune ecological niches remains a major challenge for constructing organoids tailored to studying neurological disorders.

##### Lung

In recent years, advanced lung organoid platforms have been developed from various sources including adult, fetal, and induced pluripotent stem cells, which more closely mimic the cellular structures of the airways and alveoli. The establishment of new protocols with optimized stem cell isolation and culture conditions has enabled creation of models to study the key cellular and molecular mechanisms involved in lung injury and repair. Hydrogels and patient-derived multipotent stem cells obtained through decellularization process have been successfully developed into perfused vascular organoids with patient-specific gene mutation characteristics (Tan et al. 2017; Thompson et al. 2007). By co-culturing epithelial, fibroblast, and endothelial cells in a lung microenvironment mimicking air-sac-like structures and producing lung surfactant proteins, researches have been constructed multicellular lung organoid model, such as the primitive lung-in-a-dish, and successfully reconstructed metastatic disease using primary and established cancer cells (Ramamoorthy et al. 2019). However, current research on the existing work on the vascularization of lung organoids remains limited. A significant challenge lies in constructing functional respiratory tissues that resemble the adult lung and in developing vascularized organoids with the physiological and structural features necessary to model lung diseases and development. Such advancements are essential for facilitating the discovery of emerging therapeutic strategies.

##### Heart

The ability to induce the maturation of cardiac organoids and elucidate the role of paracrine secretion in the cardiac microenvironment has been demonstrated through the construction of multicellular organoids comprising endothelial cells, pericytes, fibroblasts and cardiomyocyte (Voges et al. 2023). Stable vascularized cardiac organoids, generated by the self-assembly of vascular spheroids and cardiomyocytes, can recapitulate the pathological process such as fibrosis and dysfunction resulting from cardiac injury (Yang et al. 2024). Additionally, the generation of perfusable vascular systems using 3D chips enables the creation of personalized functional vascularized tissue scaffolds for clinical applications (Lai et al. 2021). However, given the complex demands of cardiac vascular perfusion, several challenges remain. Key issues include achieving structural maturity of the cells, inducing stochastic localization, and tailoring cellular composition and differentiation under controlled conditions. Addressing these challenges will be crucial for the continued development and application of vascularized cardiac organoids.

##### Kidney

Kidney organoid tissues generated with iPSC in combination with non-transgenic iPSC in suspension cultures exhibit extensive endothelialization and cellular features closely resembling human renal endothelium (Maggiore et al. 2024). Perfusable vascularized renal organoid chips have been shown to prompte endogenous endothelial migration into major vessels, enabling vascular integration and cellular transport (Kroll et al. 2024). Similarily, renal organoids cultured in a microfluidic system demonstrated greater sensitivity to nephrotoxic drugs, driven by fluid-flow induced shear stress (Lee et al. 2021). Despite these advancements, existing vascularized renal organoids face limitations. The lack of intercellular interactions compromises vascular stabilization and the regulation of vascular tone, and the effects of vascular network perturbations in disease states remain unclear. Future improvements in these areas will be essential to enhance the maturation and translational relevance of renal organoids.

##### Liver

Using a scalable microfluidic system, self-organized liver organoids have been shown to exhibit key functional characteristics resembling both fetal and adult liver tissue (Abbasalizadeh et al. 2023). Hepatic sinusoidal endothelial cells (LSEC) and hepatic stellate cells HSC derived from hiPSC were able to expand in vitro, displaying unique cell-specific characteristics upon induction and maturation (Koui et al. 2017). Pre-vascularization of decellularized matrices represents another approach for incorporating vasculature into the organoids. For example, using decellularized extracellular matrix scaffolds, vascularized human cortical organoids embedded in NOD-SCID immunodeficient mice successfully recruited host endothelial cells, re-establishing a functional vascular network and achieving perfusion (Shi et al. 2020). Similarly, inoculating decellularized liver scaffolds with HUVEC and hFLC cells enabled successful embedding and colonization the entire scaffold area (Baptista et al. 2011; Jin et al. 2018). Future research should focus on reducing immune rejection in vascularized organs, enhancing scalability during developmental stages, and generating specialized vascular systems and functionally adapted biliary structures.

##### Retina

Using a 3-channel microfluidic platform, endothelial, epithelial, and stromal components can be organized into a biochip that encapsulates key features of the human choroid and respond to immunomodulatory strategies (Lopez-Canosa et al. 2022). A 3D model of the retinal pigment epithelium (RPE)-capillary ciliary body (CC), constructed from iPSC-derived cells embedded in a hydrogel-based extracellular matrix, not only recapitulates key features of both healthy and AMD/MD eyes, but also allows modular control of the RPE and CC layers (Manian et al. 2021). Notably, as the retina is an extension of the central nervous system, retinal organoids can be used to investigate phenotypes of neurologically related diseases (Brighi et al. 2020; Lavekar et al. 2023; Móvio et al. 2023). Single-cell sequencing of retinal organoids has shown that their cell types mature to a stable “developmental” state in vitro at a rate comparable to that of the human retina in vivo (Cowan et al. 2020). The integration of datasets significantly enhances the potential of retinal organoids for studying disease mechanisms and developing cell-targeted therapies for repair. A critical challenge in constructing vascularized retinal organoids is achieving spatial photoreceptor alignment of retinal cell populations and successful transplantation while maintaining biocompatibility. Addressing these challenges will provide long-lasting, clinically meaningful benefits for targeted treatments.

#### Future directions in vascularized organoids

A major challenge to achieve functional vascularization of organoid is ensuring adequate perfusion. The advent of tissue-on-a-chip microfluidic systems has greatly advanced the creation of dynamic and flexible environments that mimic human biology. Custom-designed 3D-printed microfluidic chips enable hPSC-derived endothelial and perivascular cells to self-assemble into organized vascular networks. During development, vascular cell nuclei in brain-like organoids undergo physical interactions, forming integrated neurovascular-like organoids on the chip (Salmon et al. 2022).

Despite significant progress in tissue engineering and microfluidic chip technology for constructing functional blood vessels, several challenges remain. Reprogramming with iPSC is prone to introduce genetic and epigenetic abnormalities, as well as the maturity and functionality of in vitro organoids derived from hPSC remain far inferior to those of in vivo organs. Additionally, the reproducibility of 3D-printed biogels is inconsistent due to the mechanical properties of synthetic colloids, and quantifying blood flow and shear stress is critical for ensuring vascular integrity.

Future efforts should focus on reconciling the sequence of vascularization during organ construction with microenvironmental factors, as well as tailoring the generation of organ-specific vascular characteristics. Addressing these challenges will clarify the role of specialized vascularization in both healthy and diseased states, paving the way for more robust and functional vascular organoids systems.

## Conclusions and perspectives

The vascular network plays a critical role in vertebrate development, homeostasis, and regeneration. For decades, vascular biology research has focused on vascular endothelial cells because of their direct contact to the circulation system and essential function in vascular biology. Historically, blood vessels were viewed as homogeneous channels for blood flow, and vascular endothelial cells were considered a uniform, non-heterogeneous population. This concept changed until the end of twentieth century when the David Anderson and Rüdiger Klein laboratories independently identified EphrinB2 and EphB4 as molecular markers of arterial and venous endothelial cells, respectively. These discoveries revealed the molecular heterogeneity of vascular endothelial cells (Adams et al. 1999; Gerety et al. 1999; Wang et al. 1998). Shortly thereafter, the Christer Betsholtz laboratory proposed the tip-stalk cell theory in angiogenesis (Gerhardt et al. 2003), resulting in a conceptual breakthrough that significantly changed the research of angiogenesis.

The research of vascular biology across various organs revealed that endothelial cells exhibit organ-specific functions (Augustin and Koh 2017), however high-throughput comparison of vascular endothelial cells within different organs are very scarce. Shahin Rafii’s laboratory pioneered the use of microarray technology to compare endothelial cells across organs at a high-throughput level (Nolan et al. 2013). However, the limitations of microarray technology to compare gene expression in different analysis and laboratory restricted the widespread usage of these resources. With the rapid development of scRNA-seq, several labs used this approach to reveal the substantial gene expression differences between vascular endothelial cells from different organs, uncovering the extensive molecular heterogeneity of endothelial cells within the same organ or across different organs.

These findings of endothelial cell heterogeneity fundamentally reshaped the landscape of vascular biology research. In the past two decades, increasing attention has been given to arterial-venous endothelial cell differentiation and its implications for diseases such as cerebral cavernous malformations (Bourhila et al. 2024; Dulamea and Lupescu 2024). The manipulation of tip cell formation has emerged as a promising therapeutic strategy to inhibit cancer-related vascular growth, while promoting tip cell formation accelerates regeneration and repair in various organs. Despite these advances, the molecular mechanisms driving organ-specific endothelial cell differentiation remain poorly understood. Addressing this gap will require deeper analysis of scRNA-seq data to identify organ-enriched genes and the use of genetically modified mouse models to explore how these genes influence organ development, regeneration, and disease progression.

Although mouse models provide precise approach to gain insights about gene function in vascular endothelial cells, additional systems are needed to study these genes in human cells. Traditional 2D endothelial cell culture systems, such as HUVEC and HUAEC, have long been used to study vascular endothelial gene functions in the setting of human cells. However, the 2D cell culture system fails to mimic the crosstalk between endothelial cells and tissue-resident cells in the complex three-dimensional organs.

The rapid development of vascularized organoids offers a promising alternative choice. While organoids have been successfully generated for organs such as the brain and lung, their vascularization ratio remains insufficient, limiting culture duration and functional investigation. Future research should focus on improving the degree of vascularization in organoids to better mimic physiological conditions. Additionally, leveraging the knowledge of organ-specific endothelial cell heterogeneity, constructing organoids that incorporate organotypic endothelial cells could significantly enhance our ability to model the interactions between endothelial and organ-specific resident cells. This approach may provide an advanced platform for studying the function of vascular endothelial cells during human development and disease progression.

## Data Availability

Not applicable.

## Abbreviations

aCaps: Aerocyte capillaries
AMD: Age-related macular degeneration
BBB: Blood–brain barrier
CC: Capillary ciliary
CITE-seq: Cellular indexing of transcriptomes and epitopes sequencing
CNS: Central nervous system
Cyp4b1: Cytochrome P450 4B1
DLL4: Delta-like protein 4
DR: Diabetic retinopathy
Ehd3: EH domain-containing protein 3
eNOS: Endothelial nitric oxide synthase
ETS: E26 transformation specific
FTD: Frontal–temporal dementia
gCaps: General capillaries
hESC: Human Embryonic Stem Cells
hFLC: Human fetal liver cell
hiPSC: Human induced Pluripotent Stem Cells
hPSC: Human pluripotent stem cells
HUVEC: Human Umbilical Vein Endothelial Cells
iPSC: Induced Pluripotent Stem Cells
INVP: Intraneural vascular plexus
Igfbp3: Insulin-like growth factor binding protein 3
Igf1: Insulin-like growth factor 1
LSEC: Liver sinusoidal endothelial cells
MHC-II: Class II major histocompatibility complex
NO: Nitric oxide
NOTCH: Notch homolog 1
Npr3: Natriuretic peptide receptor 3
NRP-1: Neuropilin-1
PDGF: Platelet-derived growth factor
PDGFRB: PDGF receptor β
PNVP: Perineural vascular plexus
ROS: Reactive oxygen species
RPE: Retinal pigment epithelium
scATAC-seq: Single-cell ATAC-seq
scRNA-seq: Single-cell RNA sequencing
SNARE-seq: Single-nucleus chromatin accessibility and mRNA expression sequencing
snRNA-seq: Single nuclei RNA sequencing
TEC: Tumor endothelial cells
TRAP-seq: Translational ribosome affinity purification sequencing
Tspan7: Tetraspanin 7
VEC: Vascular endothelial cells
VEGF-A: Vascular endothelial cell growth factor A
2D: Two-dimensional
3D: Three-dimensional
